# Hydrocodone vs Oxycodone and Postoperative Pain and Opioid Use in Joint Arthroplasty

**DOI:** 10.1001/jamanetworkopen.2026.23079

**Published:** 2026-07-14

**Authors:** Noor A. Nahid, Natasha J. Petry, Jordan F. Baye, Chancellor F. Gray, Rachel A. Myers, Erica N. Elwood, Elizabeth C. Harris, Hrishikesh Chakraborty, Simona Volpi, Renee Rider, Paul R. Dexter, Josh F. Peterson, Todd C. Skaar, Hari K. Parvataneni, Larisa H. Cavallari, Julie A. Johnson

**Affiliations:** 1 Department of Biomedical Informatics, College of Medicine, The Ohio State University, Columbus; 2 Department of Pharmacy Practice, School of Pharmacy, North Dakota State University, Fargo; 3 Sanford Health Imagenetics, Sioux Falls, South Dakota; 4 Department of Pharmacy Practice, South Dakota State University, Brookings; 5 Florida Orthopaedic Institute, Gainesville; 6 Clinical Research Unit, Department of Medicine, Duke University School of Medicine, Durham, North Carolina; 7 Center of Pharmacogenomic and Precision Medicine, University of Florida, College of Pharmacy, Gainesville; 8 Duke Clinical Research Institute, Duke University School of Medicine, Durham, North Carolina; 9 Department of Biostatistics and Bioinformatics, Duke University, Durham, North Carolina; 10 National Human Genome Research Institute, National Institutes of Health, Bethesda, Maryland; 11 Regenstrief Institute, Indiana University, Indianapolis; 12 Department of Biomedical Informatics, Vanderbilt University Medical Center, Nashville, Tennessee; 13 Department of Medicine, Indiana University School of Medicine, Indianapolis; 14 Department of Pharmacotherapy and Translational Research and Center for Pharmacogenomics and Precision Medicine, College of Pharmacy, University of Florida, Gainesville; 15 Clinical and Translational Science Institute, The Ohio State University, Columbus; 16 Department of Internal Medicine, College of Medicine, The Ohio State University, Columbus; 17 Department of Pharmaceutics & Pharmacology, College of Pharmacy, The Ohio State University, Columbus

## Abstract

**Importance:**

Oxycodone and hydrocodone are the most frequently prescribed opioids for postoperative joint arthroplasty pain, yet comparative effectiveness data remain limited.

**Objective:**

To compare postoperative pain outcomes and opioid consumption for hydrocodone vs oxycodone among patients undergoing elective joint arthroplasty.

**Design, Setting, and Participants:**

This cohort study was a preplanned secondary analysis of a randomized clinical trial conducted among cytochrome P450 2D6 (CYP2D6) normal metabolizers (NMs) who underwent elective joint arthroplasty in 5 US surgery clinical sites from March 2021 to September 2023 and received hydrocodone or oxycodone postoperatively. Follow-up concluded in March 2024. Data analysis was conducted from October 2024 to December 2025.

**Exposure:**

Postoperative analgesia with hydrocodone or oxycodone within a multimodal pain management approach (opioids and nonopioids).

**Main Outcomes and Measures:**

Coprimary outcomes were composite pain score at postoperative day 10 (sum of current, worst, and mean pain over 7 days; range 3-15) and cumulative morphine milligram equivalents (MMEs) over 10 days. Secondary outcomes included mobility scores at day 10 and Patient-Reported Outcomes Measurement Information System anxiety and depression scores at day 30. Multivariable regressions were adjusted for demographic and clinical covariates.

**Results:**

Among 663 participants (mean [SD] age, 66 [10.6] years; 384 [57.9%] female), 217 received oxycodone and 446 received hydrocodone. Hydrocodone was associated with significantly lower composite pain scores compared with oxycodone (mean [SD], 9.0 [2.0] vs 9.3 [2.2]; adjusted *P* = .001) and significantly lower cumulative MME (mean [SD], 93.5 [159.3] vs 154.5 [121.6]; adjusted *P* < .001). Secondary outcomes did not differ between groups (mobility score: mean [SD], 26.3 [10.4] vs 30.9 [14.7]; *P* = .29; anxiety score: mean [SD], 45.4 [8.3] vs 45.5 [8.7]; *P* = .31; depression score: mean [SD], 43.8 [7.8] vs 44.0 [7.9]; *P* = .97). Findings were consistent in sensitivity analyses, restricting to opioid and acetaminophen combinations, restricting to sites prescribing both opioids, and in subgroups of knee arthroplasty, but not consistent in hip arthroplasty.

**Conclusions and Relevance:**

In this cohort study of CYP2D6 NMs undergoing joint arthroplasty, within multimodal analgesia, hydrocodone provided comparable pain control to oxycodone with significantly lower opioid exposure, supporting its use as a viable and possibly preferable analgesic option.

## Key Points

**Question:**

Is oxycodone associated with superior postoperative pain control compared with hydrocodone among cytochrome P450 2D6 (CYP2D6) normal metabolizers undergoing joint arthroplasty?

**Findings:**

In this cohort study including 663 participants receiving multimodal analgesia, hydrocodone was associated with small but significantly lower composite pain scores and significantly lower cumulative opioid consumption than oxycodone. These results were consistent across sensitivity and subgroup analyses.

**Meaning:**

These findings suggest that among CYP2D6 normal metabolizers undergoing joint arthroplasty and receiving multimodal analgesia, hydrocodone provides comparable analgesia to oxycodone with markedly lower opioid exposure.

## Teaser

This cohort study compares postoperative pain outcomes and opioid consumption for hydrocodone vs oxycodone among CYP2D6 normal metabolizers undergoing elective joint arthroplasty.

## Introduction

Nearly 1.25 million total joint arthroplasty procedures are performed annually in the US, offering patients relief from debilitating pain and a restored quality of life.^1^ Yet, the path to recovery can be paved with significant postoperative pain. Managing this pain effectively is not just a matter of comfort but crucial for enabling early mobility, facilitating rehabilitation, and preventing a cascade of complications that can delay healing.^1,2,3^

In the postoperative period, opioids remain a foundational component of pain management, with oxycodone and hydrocodone being the most frequently prescribed agents for total joint arthroplasty in the US.^4,5^ Oxycodone is more potent than hydrocodone in terms of pain relief on a milligram-to-milligram basis. Pharmacodynamic studies in both pain-free volunteers and individuals receiving or misusing prescription opioids demonstrate that oxycodone produces greater opioid effects, including miosis and subjective opioid-related effects, at equivalent doses compared with hydrocodone, with relative potency estimates suggesting oxycodone is approximately 1.5 times more potent than hydrocodone.^6,7,8^ This perception has often been carried into routine clinical practice, where many clinicians assume oxycodone will provide superior pain relief at traditional doses of the respective medications. However, when used at typical clinical doses, multiple randomized clinical trials in acute pain settings found no clinically meaningful difference in analgesic efficacy between the 2 agents.^9,10,11^ The overall evidence base for postoperative pain control, including comparative effectiveness studies comparing these 2 medications, is limited and far from conclusive.

Hydrocodone and oxycodone are both metabolized by the cytochrome P450 2D6 (CYP2D6) enzyme to form more potent metabolites (hydromorphone and oxymorphone, respectively) that have higher affinity for μ-opioid receptors than the parent compounds.^12,13,14^ However, oxycodone is pharmacologically active in its parent form, whereas hydrocodone’s analgesic effect depends more on metabolic activation.^12,13,14^ Importantly, CYP2D6 activity varies widely among individuals. Approximately 10% to 15% of individuals carry genetic variants that confer reduced or absent CYP2D6 function, termed *intermediate* or *poor metabolizer* (IM or PM, respectively) phenotypes. Additionally, many commonly prescribed medications (eg, paroxetine, fluoxetine, bupropion, duloxetine) can inhibit CYP2D6 enzymatic activity, resulting in an IM or PM phenotype and poor pain control.^15,16^

The Implementing Genomics in Practice (IGNITE) Network’s A Depression and Opioid Pragmatic Trial in Pharmacogenomics (ADOPT-PGx) trial focused on postsurgical pain and was designed to address the clinical impact of *CYP2D6* genotype–guided opioid prescribing in surgical patients.^17^ The primary ADOPT-PGx trial analysis was conducted in patients with a CYP2D6 IM and PM phenotype and demonstrated that a CYP2D6-guided approach to opioid selection did not significantly improve postoperative pain control compared with usual care in the context of multimodal analgesia.^18^ Given the size of the ADOPT-PGx study and use of both hydrocodone and oxycodone, we sought to address the comparative effectiveness of these 2 commonly used opioids in a population not impacted by pharmacogenetic variants (ie, normal metabolizers [NMs]). Therefore, we conducted a secondary analysis of the IGNITE ADOPT-PGx trial to test the hypothesis that among CYP2D6 NMs undergoing joint arthroplasty, oxycodone provides superior pain relief compared with hydrocodone in the immediate postoperative period.

## Methods

### Study Design

This cohort study was a preplanned secondary analysis of the IGNITE ADOPT PGx trial,^17^ with analysis conducted from October 2024 to December 2025, focusing on a specific patient subgroup and nonrandomized exposure comparison within the trial dataset. The parent trial was a prospective, multicenter, open-label randomized clinical trial conducted at 8 health systems in the US; patients were enrolled between March 2021 and September 2023 and followed up for 6 months after surgery. The trial protocol was approved by the Duke University Health System institutional review board (which served as the single institutional review board for all sites), and all participants provided written informed consent prior to genotyping and study procedures. The current analysis leverages deidentified data from this trial. This article adheres to the Strengthening the Reporting of Observational Studies in Epidemiology (STROBE) reporting guideline for cohort studies.

### Participants

For this study, we included participants from 5 clinical sites who were CYP2D6 NMs based on both genotype and concomitant inhibitor prescriptions, underwent major joint arthroplasty (total knee arthroplasty [TKA], total hip arthroplasty [THA], or other elective joint replacement) and were prescribed oxycodone or hydrocodone postoperatively within multimodal pain management approaches (concurrent use of opioids and ≥1 nonopioid analgesics). The remaining 3 sites had no patients undergoing arthroplasty. Individuals with chronic opioid use, defined as use on most days of the week during the past 3 months, were excluded from the trial, as defined in the study protocol and design.^17^ This study collected baseline demographic and clinical covariates that may confound postoperative pain outcomes, including age, sex, self-reported race and ethnicity, body mass index, surgery type, depression, anxiety, psychiatric disorders, diabetes, hypertension, cancer, baseline numeric pain rating, and concomitant opioid or nonopioid medication use. Race was categorized as Black, White, or other (eg, American Indian, Asian, Middle Eastern or North African or Mediterranean, Native American or Alaska Native, Native Hawaiian or Other Pacific Islander, or more than 1 race) and ethnicity was categorized as Hispanic or Latino and not Hispanic or Latino. Race and ethnicity were assessed to more comprehensively characterize the study cohort and because there are known differences in CYP2D6 functional alleles and phenotypes by continental ancestry. Variables specifically related to chronic pain were not collected because patients with chronic opioid use were excluded from the study.

### Outcomes

This study had co–primary outcomes: a composite pain score and cumulative morphine milligram equivalents (MMEs). The composite pain score was defined as the sum of current pain, worst, and mean pain in the previous 7 days (with scores ranging from 3-15 and higher scores indicating greater pain). MME was defined as postdischarge opioid consumption, with higher values indicating greater opioid consumption, normalized for number of days from discharge to data collection, as described in the previously published study.^18^ These data were collected a mean (SD) of 10 (3) days after surgery. Secondary outcomes included mobility scores, where higher scores indicate better mobility, collected 10 (3) days after surgery, and Patient-Reported Outcomes Measurement Information System (PROMIS-43) anxiety and depression scores,^19^ where higher values indicate greater symptom burden, collected a mean (SD) of 30 (7) days after surgery. Primary and secondary outcomes data were collected via a structured survey (via phone call by trained call center personnel) as part of the trial.^17^

### Statistical Analysis

The baseline and clinical characteristics are presented using means and SDs for continuous variables and frequencies with percentages for categorical variables. Continuous variables were compared between groups using the *t* test or the Wilcoxon rank-sum test, as appropriate, and categorical variables using χ^2^ or Fisher exact tests. Multivariable linear regression was used to compare primary and secondary outcomes between groups, adjusting for baseline and clinical variables that differed significantly between groups. Hospital site was not included as a confounding variable, as sites’ choice of opioid and other pain management approaches prescription were correlated. To take site into consideration, we conducted an analysis stratified by site and conducted a sensitivity analysis with sites where both opioids of interest were prescribed.

We conducted additional sensitivity and subgroup analyses to test the robustness of our findings. Some patients were prescribed tramadol in addition to the opioids of interest, so we conducted sensitivity analyses excluding patients prescribed tramadol. Because hydrocodone in the US is marketed in combination with acetaminophen, we also conducted a sensitivity analysis only in patients receiving an opioid plus acetaminophen. Some participating hospitals used 1 opioid almost exclusively. To address potential site bias and to reduce confounding by institutional protocol, we performed an analysis restricted to sites that had a mixed prescribing pattern of using both hydrocodone and oxycodone. We also performed subgroup analyses by surgery type: stratifying the cohort into patients who underwent TKA and those who underwent THA (the 2 largest subgroups). Multivariable linear regression was used to compare the outcomes between groups adjusting for the variables as described previously. All *P* values were 2-sided; *P* < .025 was considered statistically significant for co–primary outcome comparisons (corrected for 2 primary hypotheses), whereas *P* < .05 was considered statistically significant for all secondary, sensitivity, and subgroup analyses. All analyses were performed in SAS version 9.4 (SAS Institute). Data analysis was conducted from October 2024 to December 2025.

## Results

### Patient Characteristics

A total of 663 participants were included in this secondary observational analysis of the IGNITE ADOPT-PGx trial (Figure). Baseline demographics are provided in Table 1. Overall, the mean (SD) age was 66 (10.6) years, 384 participants (57.9%) were female, 80 participants (12.1%) were Black, 533 participants (80.4%) were White, and 50 participants (7.5%) identified as other race. All individuals underwent arthroplasty procedures and received either hydrocodone or oxycodone for postoperative pain management.

**Figure.**
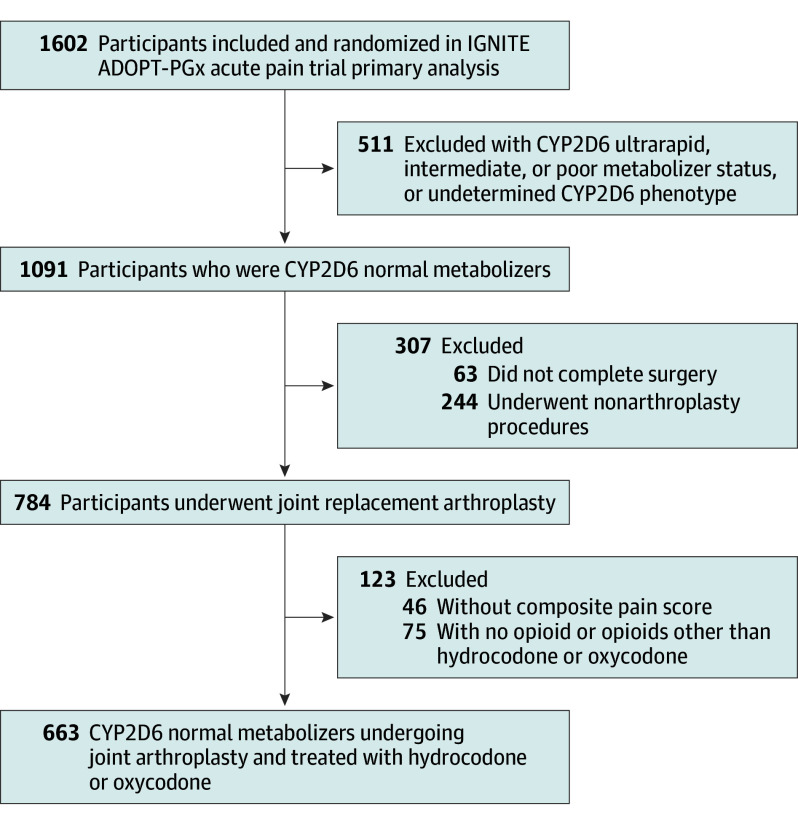
Flow Diagram of Participant Selection for the Analytic Cohort ADOPT-PGx indicates A Depression and Opioid Pragmatic Trial in Pharmacogenomics; CYP2D6, cytochrome P450 2D6; IGNITE, Implementing Genomics in Practice.

**Table 1.** Baseline Patient Characteristics

Characteristic	Patients undergoing knee, hip or other joint replacement, No. (%)		*P* value
	Oxycodone (n = 217)	Hydrocodone (n = 446)	
Age, mean (SD), y	65.5 (11.2)	66.4 (10.2)	.29
Gender^a^			
Female	128 (59.0)	256 (57.4)	.32
Male	88 (40.6)	190 (42.6)	
Race			
Black or African American	30 (13.8)	50 (11.2)	.04
White	161 (74.2)	372 (83.4)	
Other, unknown, or preferred not to answer^b^	26 (12.0)	24 (5.4)	
Ethnicity			
Hispanic or Latino	15 (6.9)	19 (4.3)	.23
Not Hispanic or Latino	202 (93.1)	427 (95.7)	
BMI, mean (SD)	32.2 (6.9)	31.9 (6.7)	.49
Medical history			
Diabetes	44 (20.3)	74 (16.6)	.24
Hypertension	117 (53.9)	228 (51.1)	.50
Depression	54 (24.9)	77 (17.3)	.02
Anxiety	47 (21.7)	85 (19.1)	.43
Psychiatric disorder	13 (6.0)	11 (2.5)	.02
Cancer	44 (20.3)	96 (21.5)	.73
Pain numeric rating, mean (SD)^c^	5.8 (2.2)	6.2 (2.1)	.34
Surgery type			
Joint replacement knee	120 (55.3)	251 (56.3)	<.001
Joint replacement hip	60 (27.6)	187 (41.9)	
Other joint replacement	37 (17.1)	8 (1.8)	
Other pain drugs			
NSAIDs	78 (35.9)	242 (54.3)	<.001
Acetaminophen	124 (57.1)	446 (100.0)	<.001
Gabapentinoid	39 (18.0)	168 (37.7)	<.001
Tramadol	83 (38.2)	31 (7.0)	<.001
Nerve block use	156 (71.9)	244 (54.7)	<.001
Site			
1	50 (23.0)	352 (78.9)	<.001
2	20 (9.2)	49 (11.0)	
3	31 (14.3)	44 (9.9)	
4	42 (19.4)	1 (0.2)	
5	74 (34.1)	0 (0.0)	

Abbreviations: BMI, body mass index (calculated as weight in kilograms divided by height in meters squared); NSAID, nonsteroidal anti-inflammatory drug.

^a^ One participant in the oxycodone group identified as other or preferred not to answer.

^b^ Other race includes individuals reporting as American Indian, Asian, Middle Eastern or North African or Mediterranean, Native American or Alaska Native, Native Hawaiian or Other Pacific Islander, or more than 1 race.

^c^ Numeric pain rating on a scale of 0-10.

Of the cohort, 371 participants (56.0%) underwent TKA, 247 participants (37.3%) underwent THA, and 44 participants (6.6%) had other joint replacement procedures. There were 217 participants (32.7%) in the oxycodone cohort and 446 participants (67.3%) in the hydrocodone cohort. Additionally, 114 participants (17.2%) received tramadol prescriptions in combination with either hydrocodone or oxycodone. The mean length of stay was 1 day, so we were able to capture nearly all postoperative opioid use. In addition to opioid therapy, 610 patients (92.0%) were also prescribed 1 of the nonopioid pain medications and 399 patients (60.2%) had a nerve block. Participants were recruited from 5 sites, and opioid (and other pain management) prescribing patterns varied substantially by site (Table 1).

### Primary and Secondary End Points

Based on differences in baseline variables (Table 1), the regression model was empirically adjusted for self-reported race and ethnicity, surgery type, depression and psychiatric comorbidities, and concomitant use of tramadol, nerve block, nonsteroidal anti-inflammatory drugs, acetaminophen, and gabapentinoids to evaluate whether postoperative pain control and MME consumption differed between hydrocodone and oxycodone. The primary outcome data and sensitivity and subgroup analysis results are shown in Table 2. In contrast with the hypothesis, the composite pain score was significantly lower in the hydrocodone cohort compared with the oxycodone cohort (mean [SD], 9.0 [2.0] vs 9.3 [2.2]; adjusted *P* = .001). The hydrocodone cohort also had significantly lower opioid use compared with the oxycodone cohort (cumulative MME: mean [SD], 93.5 [159.3] vs 154.5 [121.6]; adjusted *P* < .001). Among the secondary outcomes, mobility at day 10 (mean [SD], 26.3 [10.4] for hydrocodone vs 30.9 [14.7] for oxycodone; *P* = .29) and PROMIS-43 anxiety (mean [SD], 45.4 [8.3] vs 45.5 [8.7]; *P* = .31) and depression *T*-scores and depression scores at 30 days (mean [SD], 43.8 [7.8] vs 44.0 [7.9]; *P* = .97) were not significantly different between groups.

**Table 2.** Comparison of Primary and Secondary Outcomes Between Patients Receiving Oxycodone vs Hydrocodone for Postoperative Analgesia

Measure	Mean (SD) [No. with data]^a^		*P* value^b^	
	Oxycodone	Hydrocodone	Unadjusted	Adjusted
Co–primary outcomes				
Composite pain score at 10 d^c^	9.3 (2.2) [217]	9.0 (2.0) [446]	.09	.001
Cumulative MME at 10 d	154.5 (121.6) [208]	93.5 (159.3) [426]	<.001	<.001
Secondary outcomes				
Mobility at 10 d^d^	30.9 (14.7) [212]	26.3 (10.4) [440]	<.001	.29
PROMIS-43 anxiety at 1 mo^e^	45.5 (8.7) [209]	45.4 (8.3) [425]	.84	.31
PROMIS-43 depression at 1 mo^e^	44.0 (7.9) [209]	43.8 (7.8) [424]	.81	.97
**Sensitivity analyses**				
Excluding combination with tramadol				
Composite pain score at 10 d^c^	9.5 (2.3) [134]	9.0 (2.1) [415]	.02	.002
Cumulative MME at 10 d	131 (129.8) [125]	89.1 (162.8) [395]	.003	<.001
Only patients using acetaminophen				
Composite pain score at 10 d^c^	9.7 (2.1) [124]	9.0 (2.0) [446]	.001	.001
Cumulative MME at 10 d	170.7 (113.8) [118]	93.5 (159.3) [426]	<.001	<.001
Including sites prescribing both oxycodone and hydrocodone				
Composite pain score at 10 d^c^	9.2 (2.3) [101]	8.9 (2.0) [445]	.32	.01
Cumulative MME at 10 d	134.8 (122.8) [93]	93.2 (159.3) [425]	.006	<.001
**Subgroup analyses**				
Knee arthroplasty only				
Composite pain score at 10 d^c^	9.9 (2.1) [120]	9.3 (1.9) [251]	.009	.001
Cumulative MME at 10 d	178. 0 (131.0) [118]	92.2 (186.8) [245]	<.001	<.001
Hip arthroplasty only				
Composite pain score at 10 d^c^	9.2 (1.6) [60]	8.7 (2.1) [187]	.07	.29
Cumulative MME at 10 d	155.8 (101.3) [53]	94.2 (112.6) [173]	<.001	.50

Abbreviations: MME, morphine milligram equivalent; PROMIS-43, Patient-Reported Outcomes Measurement Information System.

^a^ Sample sizes vary across outcomes due to missing follow-up data.

^b^ Unadjusted *P* values were derived from *t* tests, and adjusted *P* values were derived from multivariable linear regression.

^c^ Range, 3-15; higher score indicates more pain.

^d^ Higher score indicates better mobility.

^e^ Higher score indicates greater symptom burden.

### Sensitivity Analyses

Results were consistent across all prespecified sensitivity analyses (Table 2). After excluding participants who received tramadol, hydrocodone remained significantly associated with lower composite pain scores (mean [SD], 9.0 [2.1] vs 9.5 [2.3]; adjusted *P* = .002) and lower cumulative MME (mean [SD], 89.1 [162.8] vs 131.0 [129.8]; adjusted *P* < .001) compared with oxycodone. In the analysis restricted to participants receiving an opioid-acetaminophen combination, findings were consistent (composite pain score: mean [SD], 9.0 [2.0] for hydrocodone vs 9.7 [2.1] for oxycodone; adjusted *P* = .001; cumulative MME: mean [SD], 93.5 [159.3] for hydrocodone vs 170.7 [113.8] for oxycodone; adjusted *P* < .001). In the analysis restricted to sites with mixed opioid prescribing (sites 1-3), the direction and significance of findings were preserved (composite pain score: mean [SD], 8.9 [2.0] for hydrocodone vs 9.2 [2.3] for oxycodone; adjusted *P* = .01; cumulative MME: mean [SD], 93.2 [159.3] for hydrocodone vs 134.8 [122.8] for oxycodone; adjusted *P* < .001) (Table 2).

### Subgroup Analyses

Among participants undergoing TKA, hydrocodone was associated with significantly lower composite pain scores (mean [SD], 9.3 [1.9] vs 9.9 [2.1]; adjusted *P* = .001) and lower cumulative MME (mean [SD], 92.2 [186.8] vs 178.0 [131.0]; adjusted *P* < .001) compared with oxycodone. Among participants undergoing THA, differences between hydrocodone and oxycodone in composite pain score (mean [SD], 8.7 [2.1] vs 9.2 [1.6]; adjusted *P* = .29) and cumulative MME (mean [SD], 94.2 [112.6] vs 155.8 [101.3]; adjusted *P* = .50) were not statistically significant, although they were directionally consistent with the primary analysis.

### Comparison Across Different Sites

Because these are nonrandomized analyses and pain management practices differed across sites, we conducted a site-level comparison of prescribing patterns, multimodal analgesic use, postoperative pain outcomes, and cumulative opioid consumption to contextualize our findings and improve clarity (Table 3). No statistical comparisons were performed for this site-level comparison. Sites are ordered from highest to lowest hydrocodone utilization, with separate summaries for patients who underwent TKA vs THA provided in eTable 1 in Supplement 1).

**Table 3.** Site-Level Comparison of Prescribing Patterns, Multimodal Analgesic Use, Postoperative Pain Outcomes, and Cumulative Opioid Consumption

Measure	Site 1 (n = 402)	Site 2 (n = 69)	Site 3 (n = 75)	Site 4 (n = 43)	Site 5 (n = 74)
Composite pain score at 10 d, mean (SD)^a^	8.9 (2.1)	9.1 (2.1)	9.8 (2.0)	10.0 (2.1)	9.0 (2.0)
Cumulative MME at 10 d, mean (SD)	83.1 (159.7)	119.5 (94.8)	193.1 (130.1)	153.6 (144.8)	180.7 (100.3)
Hydrocodone use, No. (%)	352 (87.6)	49 (71.0)	44 (58.7)	1 (2.3)	0
Oxycodone use, No. (%)	50 (12.4)	20 (29)	21 (41.3)	42 (97.7)	74 (100)
Tramadol use in addition to other opioids, No. (%)^b^	9 (2.2)	41 (59.2)	0	0	64 (86.5)
Use of any nonopioid analgesic medications, No. (%)^b^	387 (96.3)	61 (88.4)	75 (100)	31 (72.1)	55 (74.3)
Nonopioid analgesic medications used, mean (SD), No.^b^	1.9 (0.9)	1.2 (0.7)	1.8 (0.8)	1 (0.8)	1.1 (0.8)
Nerve block use, No. (%)	240 (59.7)	8 (11.6)	57 (76.0)	39 (90.7)	56 (75.7)

Abbreviation: MME, morphine milligram equivalent.

^a^ Composite pain score ranges from 3-15, higher score = more pain obtained at 10 days postoperatively.

^b^ Nonopioid analgesics included acetaminophen, nonsteroidal anti-inflammatory drugs, and gabapentinoids.

Sites 1 and 2, which had the highest hydrocodone use (87.6% and 71.0%, respectively), also had the lowest cumulative MME (83.1 and 119.5, respectively) and the best composite pain scores (8.9 and 9.1, respectively). In contrast, site 5, which prescribed oxycodone exclusively (100%), reported a similar pain score (9.0) but higher MME (180.7) where the elevated MME may be partially explained by the frequent coadministration of tramadol (86.5%). Sites 3 and 4 reported the highest pain scores (9.8 and 10.0, respectively) and lower hydrocodone use (58.7% and 2.3%, respectively). In the context of their similar pain scores, MME, and hydrocodone use, sites 1 and 2 differed notably in multimodal pain management approaches. Site 1 had the highest use of nonopioid analgesic medications (mean, 1.9 medications), with modest use of nerve block (59.7%), while site 2 had among the lowest use of nonopioid analgesics (mean, 1.2 medications) and the lowest use of nerve block (11.6%). We examined site-level pain scores and cumulative MME by opioid group and observed that across sites where both opioids were prescribed, hydrocodone was consistently associated with comparable composite pain scores at lower cumulative MME compared with oxycodone, supporting the overall findings.

## Discussion

In this cohort study of postoperative analgesia in CYP2D6 NMs undergoing joint arthroplasty, we observed that patients prescribed hydrocodone had lower composite pain scores over the 10-day postoperative period, and their total opioid consumption (in MME) was significantly lower, compared with patients prescribed oxycodone. Therefore, we rejected our initial hypothesis that oxycodone provides better pain control than hydrocodone. The findings were consistent across multiple sensitivity analyses and were especially pronounced in the TKA subgroup, which typically reports greater pain than THA.

Although the difference in composite pain score between the hydrocodone and oxycodone groups was statistically significant, the absolute difference was small. Therefore, this difference alone may not represent a clinically meaningful improvement in pain.

While there is a common clinical belief that oxycodone provides superior pain control to hydrocodone, this is not well-supported in literature. A double-blind randomized trial with 220 participants found no clinically or statistically significant difference between hydrocodone/acetaminophen 5 mg/325 mg and oxycodone/acetaminophen 5 mg/325 mg for treating acute extremity pain, with both opioids reducing pain by approximately 50% and yielding nearly identical patient satisfaction.^10^ A separate randomized trial in emergency department patients with acute extremity pain found no significant differences in pain reduction at 2 hours among single-dose treatment with ibuprofen and acetaminophen vs 3 different opioids-acetaminophen combinations, including oxycodone, hydrocodone, and codeine.^11^ Several other trials and observational studies reported pain outcomes and satisfaction between the 2 opioids at usual doses.^9,20,21^ One of the limitations of these prior studies is failure to consider the impact of *CYP2D6* phenotypes while looking at the efficacy of hydrocodone and oxycodone,^9,10,11,21^ where inclusion of PMs or IMs in the study could bias the results toward oxycodone, given that its analgesic effect is less dependent on CYP2D6.^22^ This is a strength of our study, as we restricted the analysis to CYP2D6 NMs. By including only NMs, we ensured that the analyses were not confounded by metabolic activation of hydrocodone and oxycodone, increasing the confidence that the observed differences in pain outcomes were due to the drugs’ intrinsic analgesic efficacy.^12,23^

Across sites, higher utilization of hydrocodone was directionally associated with lower opioid consumption and comparable or better pain control, consistent with overall findings. Although multimodal approaches could influence these outcomes, use of these other analgesics and nerve blocks were statistically controlled for in the analysis. Sites 1 and 2, which had the highest hydrocodone use, showed the lowest MME and the best pain scores, despite having the lowest use of nerve block. In contrast, site 5, where oxycodone was exclusively used vs hydrocodone, pain control was comparable (particularly among patients who underwent THA) but required more than 2-fold the MME of site 1. Overall, site-level prescribing patterns from multiple angles consistently suggest that oxycodone provides similar or worse pain control than hydrocodone with substantially higher opioid exposure.

It is notable that we excluded patients with impaired CYP2D6 metabolism based on either genotype or drug interactions, in which hydrocodone is expected to have reduced efficacy. It is possible that in CYP2D6 IMs or PMs, oxycodone would be the preferred therapy, although the primary results of the ADOPT-PGx trial secondary analyses did not support that a genotype-guided approach to postoperative pain management improves pain control.^18^

Our findings have practical implications for postoperative pain management, especially in the context of personalized medicine and opioid-sparing strategies. For patients who are CYP2D6 NMs, a multimodal approach with hydrocodone appears to be a safe and effective option for managing acute post–total joint arthroplasty pain, achieving pain relief that was comparable to or better than oxycodone, with a lower MME. Given the ongoing effort to reduce opioid prescriptions and doses to mitigate risks of adverse effects, opioid persistence, and opioid use disorder,^18^ the ability to manage pain with a lower opioid burden is highly valuable.

### Strengths and Limitations

Key strengths of this study include its large sample size and design reflecting typical postoperative pain management scenarios, with its inclusion of multiple hospitals and practice patterns improving external validity and applicability if the CYP2D6 metabolizer status is known. MME data were gathered by trained call center personnel confirming exact prescription and the doses the patients had used vs an unreliable delayed recall, self-reported method. It also relied on patient-reported outcomes of pain, providing a valuable patient-centric context as an outcome. This study rigorously controlled for potential confounders, including genotype stratification, adjusting for differences in baseline factors and pain management differences. Additionally, multiple sensitivity analyses consistently supported the primary finding. By examining both pain scores and MME together, our study provides a holistic view of analgesia quality (pain relief achieved) in relation to MME. Furthermore, to our knowledge, this is one of the first comparative studies of hydrocodone vs oxycodone in a postoperative pharmacogenomics context, representing novel contribution to genotype-guided therapy literature.

This study has several limitations. As a secondary analysis, opioid assignment was not randomized to hydrocodone vs oxycodone. Unmeasured confounders, including surgeon preference, anticipated pain severity, patient preference for opioid choice, unrecorded patient factors or chronic conditions, and other clinical considerations, could still exist despite adjusting for measured confounders and stratifying by site and surgery. Site 1 was the largest enroller and highest user of hydrocodone, so unrecognized or implicit local pain management protocols may impact outcomes. We addressed potential confounding through multivariable regression analysis; propensity scoring, although not performed here, produced similar results in a prior analysis.^22^ Pain scores were patient reported, introducing subjective bias and potential inaccuracy in recall, although use of the composite pain score may mitigate this. This trial captured nerve block use as a binary variable (yes or no) without specifying block type. As this was a secondary analysis of a clinical trial and was restricted to CYP2D6 NMs, the findings may not be fully generalizable to the broader postoperative population receiving hydrocodone. However, the primary outcome data from the IGNITE ADOPT-PGx trial do not suggest that the CYP2D6 phenotype has a clinically relevant impact on postoperative pain outcomes in the context of multimodal pain management approaches. One of our co–primary outcome variables was MME, which provides a useful and widely accepted standardized measure for comparing opioid exposure across different opioids. However, as with any conversion-based metric, it may not fully capture interindividual pharmacodynamic variability or the contribution of active metabolites. Therefore, the lower MME observed in the hydrocodone group should be interpreted as reflecting lower estimated opioid exposure based on standard conversion factors, rather than as a precise pharmacodynamic equivalence measure.

## Conclusions

In this cohort study of CYP2D6 NMs undergoing joint arthroplasty within multimodal analgesic therapy, hydrocodone was associated with equivalent or better pain control than oxycodone, with significantly lower opioid exposure. Consistent findings across sensitivity analyses and subgroups supported hydrocodone as a viable and possibly preferable option for acute postoperative pain management in this setting.

## Notes

Supplement 1. **eTable.** Site-level comparison of prescribing patterns, multimodal analgesic use, postoperative pain outcomes, and cumulative opioid consumption among total knee arthroplasty and total hip arthroplasty patients

Supplement 2.
The IGNITE Investigators

Supplement 3.
Data Sharing Statement
