# Supplementary material for: Hydrocodone vs Oxycodone and Postoperative Pain and Opioid Use in Joint Arthroplasty
Source: JAMA Netw Open. 2026 Jul 14;9(7):e2623079. doi: 10.1001/jamanetworkopen.2026.23079 (PMC13370303; doi:10.1001/jamanetworkopen.2026.23079)
Supplement: Supplement 1. — eTable. Site-level comparison of prescribing patterns, multimodal analgesic use, postoperative pain outcomes, and cumulative opioid consumption among total knee arthroplasty and total hip arthroplasty patients [file jamanetwopen-e2623079-s001.pdf]

Supplemental Online Content

Nahid NA, Petry NJ, Baye JF, et al; the IGNITE Investigators. Hydrocodone vs oxycodone and postoperative pain and opioid use in joint arthroplasty. *JAMA Netw Open*. 2026;9(7):e2623079. doi:10.1001/jamanetworkopen.2026.23079

eTable. Site-level comparison of prescribing patterns, multimodal analgesic use, postoperative pain outcomes, and cumulative opioid consumption among total knee arthroplasty and total hip arthroplasty patients

This supplemental material has been provided by the authors to give readers additional information about their work.

© 2026 Nahid NA et al. *JAMA Network Open*.

eTable. Site-level comparison of prescribing patterns, multimodal analgesic use, postoperative pain outcomes, and cumulative opioid consumption among total knee arthroplasty (TKA) and total hip arthroplasty (THA) patients.

	Site 1	Site 2	Site 3	Site 4	Site 5
**Total knee arthroplasty only**					
	**n= 236**	**n= 32**	**n= 30**	**n= 30**	**n= 43**
**Baseline pain score**	5.9	6.2	6.1	6.3	4.7
**Composite pain score at 10 days**	9.2	9.7	10.6	10.1	9.7
**Cumulative MME at 10 days**	93.6	113.9	171.8	173.3	197.6
**Hydrocodone use, %**	92.0%	71.9%	33.3%	3.3%	0.0%
**Oxycodone use, %**	8.0%	28.1%	66.7%	96.7%	100.0 %
**Tramadol use in addition to other opioids, %**	2.5%	58.4%	0.0%	0.0%	86.1%
**Use of any non-opioid analgesic medications, %**	97.5%	90.6%	100.0%	70.0%	67.4%
**Average number of non-opioid analgesic medications used**	1.9	1.3	1.7	0.9	1
**Nerve block use, %**	82.6%	3.1%	73.3%	90.0%	93.0%
**Total hip arthroplasty only**					
	**n= 144**	**n= 30**	**n= 44**	**n= 12**	**n= 17**
**Baseline pain score**	6.5	6.4	6.8	7.5	5.6
**Composite pain score at 10 days**	8.6	9	9.2	9.8	8.8
**Cumulative MME at 10 days**	71	136.3	207.3	112.5	180
**Hydrocodone use, %**	92.4%	70.0%	75.0%	0.0%	0.0%
**Oxycodone use, %**	7.6%	30.0%	25.0%	100.0%	100%
**Tramadol use in addition to other opioids, %**	2.1%	66.7%	0.0%	0.0%	94.1%
**Use of any non-opioid analgesic medications, %**	97.2%	90.0%	100.0%	83.3%	76.5%
**Average number of non-opioid analgesic medications used**	2	1.1	1.9	1.3	1.2
**Nerve block use, %**	18.1%	0.0%	79.6%	100.0%	23.5%

Non-opioid analgesics: acetaminophen, NSAIDs, gabapentinoid

© 2026 Nahid NA et al. *JAMA Network Open*.
