# Supplementary material for: Hydrocodone vs Oxycodone and Postoperative Pain and Opioid Use in Joint Arthroplasty
Source: JAMA Netw Open. 2026 Jul 14;9(7):e2623079. doi: 10.1001/jamanetworkopen.2026.23079 (PMC13370303; doi:10.1001/jamanetworkopen.2026.23079)
Supplement: Supplement 2. — The IGNITE Investigators [file jamanetwopen-e2623079-s002.pdf]

Supplemental Online Content: Nonauthor Collaborators
*First name, last name, and suffix (if applicable) are required and will appear in PubMed.

| *Group Name(s): IGNITE Pragmatic Trials Network | | | | | | | |
|---|---|---|---|---|---|---|---|
| ***First Name and Middle Initial(s)** | ***Last Name** | ***Suffix (eg, Jr, III)** | Academic Degrees | Institution | Location (city, state/province, country) | Role or Contribution, eg, chair, principal investigator | Group (if more than 1 Group listed in the byline) and/or Subgroup (eg, Steering Committee) |
| Neil | Calman | | | | | | |
| Kerri | Cavanaugh | | | | | | |
| Geoff | Ginsburg | | | | | | |
| Kady-Ann | Steen-Burrell | | | | | | |
| Bhargav | Adagarla | | | | | | |
| Sarah | George | | | | | | |
| Phyllis | Kennel | | | | | | |
| Kristen | Linney | | | | | | |
| Rania | Metry | | | | | | |
| Wanda | Parker | | | | | | |
| Gayle | Passmore | | | | | | |
| Carol | Pereira | | | | | | |
| Teji | Rakhra-Burris | | | | | | |
| Jaclyn | Holland | | | | | | |
| Yashika | Johnson | | | | | | |
| Ryanne | Wu | | | | | | |
| Ruth | Lehan | | | | | | |
| Tyffany | Locklear | | | | | | |
| Sabrina | Clermont | | | | | | |
| Bart | Ferket | | | | | | |
| Kenneth | Fifer | | | | | | |
| Diane | Hauser | | | | | | |
| Joseph | Kannry | | | | | | |
| James | Murrough | | | | | | |
| Janet | Seo | | | | | | |
| Tatiana | Sabin | | | | | | |
| Nandini | Shroff | | | | | | |
| Saskia | Shuman | | | | | | |
| Abi | Colwell | | | | | | |
| Amy M. | Breman | | | | | | |

Supplemental Online Content: Nonauthor Collaborators
*First name, last name, and suffix (if applicable) are required and will appear in PubMed.

| ***First Name and Middle Initial(s)** | ***Last Name** | ***Suffix (eg, Jr, III)** | Academic Degrees | Institution | Location (city, state/province, country) | Role or Contribution, eg, chair, principal investigator | Group (if more than 1 Group listed in the byline) and/or Subgroup (eg, Steering Committee) |
|---|---|---|---|---|---|---|---|
| Zeru | Desta | | | | | | |
| Cathy | Fulton | | | | | | |
| Jennelle C. | Hodge | | | | | | |
| Sheryl | Lynch | | | | | | |
| Jonathan | Oliver | | | | | | |
| Victoria M. | Pratt | | | | | | |
| Ross | Robinson | | | | | | |
| Elizabeth | Rowe | | | | | | |
| Jennifer | Stuart | | | | | | |
| Ashely | Vetor | | | | | | |
| Kelsey | Cook | | | | | | |
| Karam | Diaby | | | | | | |
| Julio | Duarte | | | | | | |
| Ben | Duong | | | | | | |
| Karla | Giron | | | | | | |
| Elizabeth | Eddy | | | | | | |
| Julia | Krutov | | | | | | |
| Carol | Mathews | | | | | | |
| Haesuk | Park | | | | | | |
| Robyn | Nelson | | | | | | |
| Ryan | Rhoden | | | | | | |
| Caroline | Schlierle | | | | | | |
| Taylor | Sullivan | | | | | | |
| Almut | Winterstein | | | | | | |
| Kristen | Wiisanen | | | | | | |
| Sara | Van Driest | | | | | | |
| Sara | Block | | | | | | |
| Michelle | Benck | | | | | | |
| Chantel | Bender | | | | | | |
| Sylvia | Eluhu | | | | | | |
| Colette | Free | | | | | | |
| Shane | Gonnelly | | | | | | |

Supplemental Online Content: Nonauthor Collaborators
*First name, last name, and suffix (if applicable) are required and will appear in PubMed.

| ***First Name and Middle Initial(s)** | ***Last Name** | ***Suffix (eg, Jr, III)** | Academic Degrees | Institution | Location (city, state/province, country) | Role or Contribution, eg, chair, principal investigator | Group (if more than 1 Group listed in the byline) and/or Subgroup (eg, Steering Committee) |
|---|---|---|---|---|---|---|---|
| Sarah | Hedeen | | | | | | |
| Steven | Houtschilt | | | | | | |
| Michelle | Liu | | | | | | |
| Salisha | Marryshow | | | | | | |
| Nicole | Neville | | | | | | |
| Natasha | Petry | | | | | | |
| Sidd | Pratap | | | | | | |
| Vernon | Sherden | | | | | | |
| Kimberly | Snell | | | | | | |
| Ana | Tomescu | | | | | | |
| Megan | Trietsch | | | | | | |
| James | Walker | | | | | | |
| Deanna | Web | | | | | | |
| Erin | Whiting | | | | | | |
| Russell A. | Wilke | | | | | | |
| Sarah | Hutchison | | | | | | |
| Natalie | Kucher | | | | | | |
| Ebony | Madden | | | | | | |
| Jessica | Reinach | | | | | | |
| Ismail | Safi | | | | | | |
| Ella | Samer | | | | | | |
| Lani | Banez | | | | | | |
| Lisa | Bendz | | | | | | |
| Jackie | Jordan | | | | | | |
| Ann | McGee | | | | | | |
| Christina | Nix | | | | | | |
| Lorraine | Vergara | | | | | | |
| Tyler | Colon | | | | | | |
| Aaishah | Francis | | | | | | |
| Najiba | Khan | | | | | | |
| Camila | Tan Lam | | | | | | |
| Michelle | Sciarrino | | | | | | |

Supplemental Online Content: Nonauthor Collaborators
*First name, last name, and suffix (if applicable) are required and will appear in PubMed.

| ***First Name and Middle Initial(s)** | ***Last Name** | ***Suffix (eg, Jr, III)** | Academic Degrees | Institution | Location (city, state/province, country) | Role or Contribution, eg, chair, principal investigator | Group (if more than 1 Group listed in the byline) and/or Subgroup (eg, Steering Committee) |
|---|---|---|---|---|---|---|---|
| Emma | Maiman-Stadtmauer | | | | | | |
| Sofia | Medina-Pardo | | | | | | |
| Ololade (Lola) | Williams | | | | | | |
| Sheng | Luo | | | | | | |
| Kevin | Anstrom | | | | | | |
| Alicia | Ellis | | | | | | |
| Stephen | Ellis | | | | | | |
| Hwasoon | Kim | | | | | | |
| Peter | Merrill | | | | | | |
| Rachel | Myers | | | | | | |
| Lilin | She | | | | | | |
| Jennifer | Shepherd | | | | | | |
| Saira | Siddiqui | | | | | | |
| Jun | Wen | | | | | | |
| John | Howard | | | | | | |
| Alia | Jamison | | | | | | |
| Lydia | Joyner | | | | | | |
| Ellie | Kiihne | | | | | | |
| Libbie | Silverman | | | | | | |
| Jordan | Sonneville | | | | | | |
| Laurel | Tokar | | | | | | |
| Daviana | Buck | | | | | | |
| Nicole | Canchucaja | | | | | | |
| Porshia | Cook | | | | | | |
| Ariel | Jacobs | | | | | | |
| Danielle | John | | | | | | |
| Samantha | Guagliardo | | | | | | |
| Mirta (Mimi) | Milanes | | | | | | |
| Wambui | Ngari | | | | | | |
| Megan | Triestch | | | | | | |
| Abraham | Garcia | | | | | | |

Supplemental Online Content: Nonauthor Collaborators
*First name, last name, and suffix (if applicable) are required and will appear in PubMed.

| ***First Name and Middle Initial(s)** | ***Last Name** | ***Suffix (eg, Jr, III)** | Academic Degrees | Institution | Location (city, state/province, country) | Role or Contribution, eg, chair, principal investigator | Group (if more than 1 Group listed in the byline) and/or Subgroup (eg, Steering Committee) |
|---|---|---|---|---|---|---|---|
| Carol | Gutierrez | | | | | | |
| Maria | Lopez | | | | | | |
| Alan | Mejia | | | | | | |
| Anaite | Montes Bu | | | | | | |
| Deanna | Webb | | | | | | |
| Sonja | White | | | | | | |
| Ali | Brewer | | | | | | |
| Brittni | Deadrick | | | | | | |
| Alexis | Meyer | | | | | | |
| Blair | Svennes | | | | | | |
| Hana | Al Alshaykh | | | | | | |
| Tala | Basha | | | | | | |
| Emily | Cicali | | | | | | |
| Asia | Cobb | | | | | | |
| Brendon | Cooper | | | | | | |
| Amanda | Elchynski | | | | | | |
| Amanda | Elsey | | | | | | |
| Anuksha | Gotmare | | | | | | |
| Lauren | Lemke | | | | | | |
| Arielle | Nelson | | | | | | |
| Michael | Read | | | | | | |
| Genevieve | Rosier | | | | | | |
| Isabella | Schultz | | | | | | |
| Chiara | Chermont Spina | | | | | | |
| Joshua | Terrell | | | | | | |
| Alexa | Valko | | | | | | |
| Precious | Williams | | | | | | |
| Tracy | Leonard | | | | | | |
| David | Smith | | | | | | |
| Marian | Davis | | | | | | |
| Vincent | Guerra | | | | | | |
| Jennifer | Salo | | | | | | |

Supplemental Online Content: Nonauthor Collaborators
*First name, last name, and suffix (if applicable) are required and will appear in PubMed.

| ***First Name and Middle Initial(s)** | ***Last Name** | ***Suffix (eg, Jr, III)** | Academic Degrees | Institution | Location (city, state/province, country) | Role or Contribution, eg, chair, principal investigator | Group (if more than 1 Group listed in the byline) and/or Subgroup (eg, Steering Committee) |
|---|---|---|---|---|---|---|---|
| Claudia | Cartaya Torres | | | | | | |
| Jorge | Valedon | | | | | | |
| Paola | Nieves Viana | | | | | | |
| Alexander | Parker | | | | | | |
| Jose | Alonso | | | | | | |
| Jeffery | Gainer | | | | | | |
| Morgan | Howard | | | | | | |
| Fatoumata | Kaba | | | | | | |
| Brianna | Rivers | | | | | | |
| Liliana | Serrano | | | | | | |
| Kimberly | Vigal | | | | | | |
| ADeia | Williams | | | | | | |
| Angelo | Lazaro | | | | | | |
| Salisha | Merryshow | | | | | | |
| Henry | Ong | | | | | | |
| Asiyanbola | Bolanle | | | | | | |
| John Thomas | Callaghan | | | | | | |
| Justin | Deen | | | | | | |
| Michael T. | Eadon | | | | | | |
| Daniel | Getu | | | | | | |
| Blake | Goff | | | | | | |
| Joseph | King | | | | | | |
| Eric A. | Larson | | | | | | |
| Brandi | Plunkett | | | | | | |
| Luis | Pulido | | | | | | |
| Marc B. | Rosenman | | | | | | |
| Robert | Van Demark, Jr | | | | | | |
| Walter W. | Virkus | | | | | | |
| Thomas | Wright | | | | | | |
| Jonathan | Wright | | | | | | |
