# Supplementary material for: Hydrocodone vs Oxycodone and Postoperative Pain and Opioid Use in Joint Arthroplasty
Source: JAMA Netw Open. 2026 Jul 14;9(7):e2623079. doi: 10.1001/jamanetworkopen.2026.23079 (PMC13370303; doi:10.1001/jamanetworkopen.2026.23079)
Supplement: Supplement 3. — Data Sharing Statement [file jamanetwopen-e2623079-s003.pdf]

Data Sharing Statement

Nahid. Hydrocodone vs Oxycodone and Postoperative Pain and Opioid Use in Joint Arthroplasty. *JAMA Netw Open*. Published July 14, 2026.
doi:10.1001/jamanetworkopen.2026.23079

Data
Data available: Yes
Data types: Deidentified participant data
How to access data: Deidentified data and data dictionaries are available via the National Human Genome Research Institute (NHGRI) Genomic Analysis, Visualization and Informatics Lab-space (AnVIL) (https://anvilproject.org/), PHS#: ADOPT PGx Acute Pain Trial: phs004058.v1.p1.
When available: beginning date: 03-15-2026

Supporting Documents
Document types: None

Additional Information
Who can access the data: Data will be made available to qualified researchers who apply through the NHGRI AnVIL platform and receive approval for access.
Types of analyses: All analyses must comply with NIH genomic data-sharing policies and AnVIL's data-use requirements.
Mechanisms of data availability: Data will be made available through the NHGRI AnVIL platform and after receiving approval for access.
